# Quality Assessment and Factor Analysis of Systematic Reviews and Meta-Analyses of Endoscopic Ultrasound Diagnosis

**DOI:** 10.1371/journal.pone.0120911

**Published:** 2015-04-23

**Authors:** Danlu Liu, Jiaxin Jin, Jinhui Tian, Kehu Yang

**Affiliations:** 1 The First Clinical Medical College of Lanzhou University, Lanzhou, Gansu, China; 2 Evidence Based Medicine Center, School of Basic Medical Science of Lanzhou University, Lanzhou, Gansu, China; 3 Key Laboratory of Evidence Based Medicine and Knowledge Translation of Gansu Province, Lanzhou, Gansu, China; 4 The Second Clinical Medical College of Lanzhou University, Lanzhou, Gansu, China; Universitat Wien, AUSTRIA

## Abstract

**Background:**

Comprehensive monitoring of the quality of systematic reviews (SRs) and meta-analyses (MAs) of endoscopic ultrasound (EUS) requires complete and accurate reporting and methodology.

**Objective:**

To assess the reporting and methodological quality of SRs/MAs on EUS diagnosis and to explore the potential factors influencing articles’ quality.

**Methods:**

The quality of the reporting and methodology was evaluated in relation to the adherence of papers to the PRISMA checklist and the AMSTAR quality scale. The total scores for every criterion and for every article on the two standards were calculated. Data were evaluated and analyzed using SPSS17.0 and RevMan 5.1 in terms of publication time, category of reviews, category of journals, and funding resource.

**Results:**

A total of 72 SRs/MAs was included, but no Cochrane Systematic Reviews (CSRs) were obtained. The number of SRs/MAs ranged from 1 in 1998 to 15 in 2013; 88.1% used the QUADAS tool; the average overall scores by PRISMA statement and AMSTAR tool were 19.9 and 5.4, respectively. Scores on some items showed substantial improvement after publication of PRISMA and AMSTAR. However, no reviews followed the criterion of protocol and registration, and only 11.1% of articles fulfilled the criterion of literature search. SRs/MAs from the Science Citation Index (SCI) were of better quality than non-SCI studies. Funding resource made no difference to quality. Regression analysis showed that time of publication and inclusion in the SCI were significantly correlated with total scores on the two standards.

**Conclusion:**

The reporting and methodological quality of SRs/MAs on EUS diagnosis has improved measurably since PRISMA and AMSTAR checklists released. It is hoped that CSR in this field will be produced. Literature searching and protocol criteria, as well as QUADAS-2 tool need to be addressed more in the future. Time of publication and SCI relate more to the overall quality of SRs/MAs than does funding resource.

## Introduction

Endoscopic ultrasound (EUS) is a rapidly developing technique, which fully takes advantage of both endoscopy and ultrasonography. Over the last two decades, it has been widely applied in initial diagnosis and effective therapy in a wide spectrum of patients [1]. Compared with other diagnostic tests such as CT, MRI and ERCP, EUS provides an excellent complement in preoperative diagnosis and staging of tumors especially in pancreatic malignancy [2,3]. Moreover, on its foundation, fine-needle aspiration (FNA) has been widely used in the diagnosis of tumor tissues [3].

In the age of evidence-based medicine, systematic reviews(SRs)/meta-analyses(MAs) were developing rapidly and recognized as a higher level of evidence than other research types for issues of evidence-based medicine [4], particularly in the Cochrane systematic review (CSR), which was famous for its rigorous methodology [5,6]. An increasing number of SRs/MAs had emerged to assist clinicians in making evidence-based decisions, but clinicians and investigators lacked confidence to use the evidence especially systematic review of diagnostic test accuracy [7].

With the rapid development of advanced techniques, a number of standards were produced one after another, attempting to solve problems of research quality in recent years [8–10]. It is known that inadequate reporting can lead to false credence and biased results, thus misleading clinicians. [11]. Consequently, PRISMA (Preferred Reporting Items for Systematic Review and Meta-Analyses) statement was developed in 2009 after elaborative revision [8,11], providing a standardized framework to help authors report their findings in a complete and transparent manner. Ever since its release, more and more researchers have utilized it to assess SRs/MAs’ reporting quality, yet some special subtle differences were not shown in diagnostic reviews [12]. Meanwhile, the assessment of methodological quality was also a key step in relation to its reliability and validity [13]. Among all currently existing methodological quality assessment tools, AMSTAR (Assessment of Multiple Systematic Reviews) was considered a reliable and valid measurement tool [14,15]. The instrument had been developed over a decade based on empirical evidence, expert consensus and previous tools such as Overview Quality Assessment Questionnaire (OQAQ, 1991)[15]. Regarded as the best quality assessment tool of SRs/MAs by CADTH (The Canadian Agency for Drugs and Technologies in Health), it involves novel ideas in flow diagram, publication bias and language restriction [16].

Recently, the two standards have been widely accepted and utilized due to their reliability and reproducibility [17,18], but the reporting and methodological quality of SRs/MAs on EUS diagnosis was still unclear around the world. Therefore, this study aims to evaluate their qualities and explore the potential factors influencing the articles’ quality such as publication time, category of reviews, category of journals and funding resources.

## Methods

### Literature search

A comprehensive literature search was conducted in multiple databases including PubMed (1966~2013.7), Web of Science (1980~2013.7), The Cochrane Library (~2013.7), EMBASE.com (1974~2013.7), Chinese Biomedical Literature Database (CBM, 1978~2013.7), China National Knowledge Infrastructure (CNKI, 1994~2013.7) and Wanfang Database (1997~2013.7). Our search terms included “endoscopic ultrasound”, “endosonography”, “ultrasongraphy”,“systematic review” and “meta-analysis”. The syntaxes were adjusted corresponding to different database. Details of the search strategies were in S1 Text of Supporting information. The reference lists of all articles selected for the review were screened for potentially relevant articles not identified by the initial search. The electronic search was complemented by a hand search of related journals to ensure that all eligible studies were captured.

### Inclusion criteria and study selection

To be eligible, SRs/MAs of diagnostic studies on EUS, which is defined as a technology to distinguish between patients with and without disease, were included, while conference abstracts, case reports and dissertations about the accuracy of EUS were excluded. No language was restricted.

Two investigators (Liu DL and Jin JX) independently reviewed titles and abstracts. Diagnostic reviews often reported a number of diagnostic effect index to describe review’s accuracy such as sensitivity, specificity, ±LR,±PV, DOR, AUC or accuracy and the highest frequency is “sensitivity”, “specificity” or “accuracy”. Thus, the full texts were remained to exclude the reviews without mentioning the term “accuracy” or “sensitivity” or “specificity”. Disagreements were resolved by discussion.

### Quality assessment

The PRISMA statement with a 27-point checklist has been a reliable tool commonly used to evaluate the overall reporting quality of SRs/MAs [9,16]. To assess the degree of compliance, every item was rated as “yes” for total compliance, “unclear” for partial compliance or”no” for non-compliance, corresponding to the score values of ‘1’, ‘0.5’ or ‘0’, respectively. In addition to reporting criteria, AMSTAR tool, as a generic methodological standard, has been used to evaluate the quality of research. By contrast with global assessment, AMSTAR has been well received with a good practicability [13]. It consisted of 11 points and the terms of “yes”, “no”, “can’t answer” and “not applicable” were used to assess these criteria. Then the total scores for every criterion and for every article on the two standards were calculated.

### Data extraction and data analysis

Data was extracted and listed in predesigned table, including publication time, study type, category of target disorder, first author country, category of journals and funding resource. Considering that the endorsement of PRISMA resulted in increases of reporting and methodological quality [19], we had an interest in investigating instruction for authors of included journals to evaluate the endorsement of PRISMA.

Two investigators (Liu DL and Jin JX) then independently extracted data in terms of the above items. A third investigator (Tian JH) was responsible for adjudicating any disagreements until a consensus was reached. As PRISMA statement was released in 2009 and AMSTAR tool in 2007, articles were divided into groups by the publication time. CSR has gone through burgeoning development due to rigorous management and robust registration platform [5,17]. The distinctiveness of Science Citation Index (SCI) in category of journals and funding resources may result in a significant difference. Thus, the factors of subgroup analysis were presented as follows: publication time (≥2009 vs. ≤2008 in PRISMA; ≥2007 vs. ≤2006 in AMSTAR), category of reviews (CSR vs. Non-CSR), category of journals (SCI vs. Non-SCI) and funding resources (Fund vs. Non-Fund).

According to these subgroups, each article was classified and the data was analyzed using SPSS 17.0 and RevMan 5.1, respectively. A *p* value ≤0.05 indicated a statistically significant difference, which was further assessed by visual inspection of a forest plot and 95% CI for the pooled quality assessment.

## Results

### Literature search

**Fig 1:**
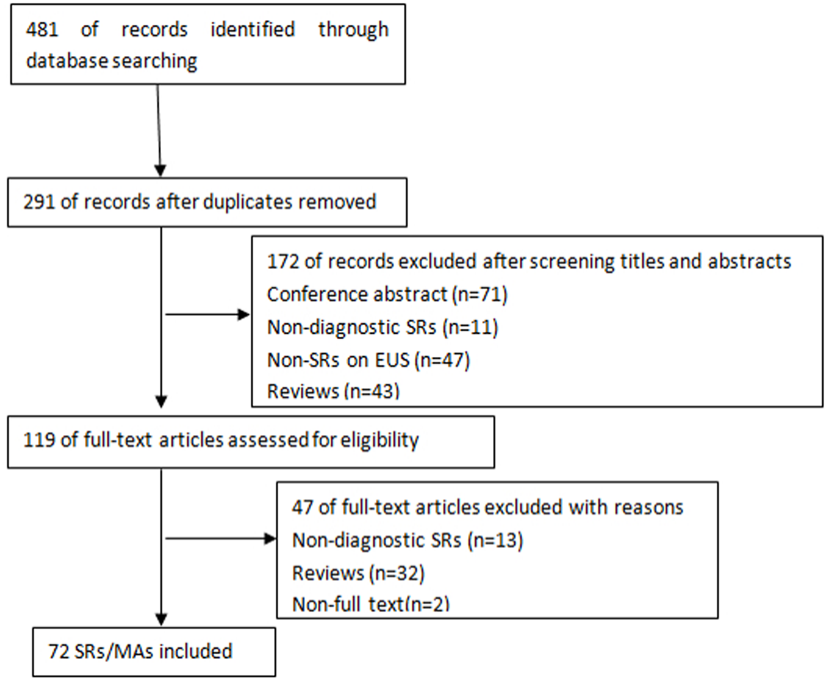
Flow chart for the literature search.

The original search yielded 481 results (Fig 1) and 291 studies were retained through duplicating. A further 172 studies were excluded by abstract review and the remaining 119 articles were reviewed in depth. By intensive screening of full texts, reference lists of included paper, and hand search, 13 non-diagnosis articles and 34 reviews were excluded. Finally, 72 articles were discussed in this study. See in S2 Text of Supporting information.

### Characteristics of Included Studies

The 72 reviews were extracted from 41 journals and the majority of journals (36.6%, 15/41) were from the United States. The two journals of the highest frequency were *Gastrointestinal Endoscopy* (24.4%, 10/41), and *Digestive Diseases and Sciences* (14.6%, 6/41). There were 33 journals from Science Citation Index (SCI) with IF varying from 1 to 30 (the highest was JAMA, IF: 30.387 in 2013), and the IF of the majority (75.8%, 25/33) ranged from 2 to 6. After reviewing all these journals, only one-fifth (9/41) of them endorsed PRISMA in instructions for authors including PRISMA flow diagram and checklist.

Table 1 summarizes the characteristics of all SRs/MAs in the field of EUS diagnosis. Of all the 72 articles, 90.3% (65/72) were written in English and 9.7% (7/72) in Chinese. Articles from SCI journals accounted for 87.5% (63/72) and 11 (15.3%) articles had funds support mainly from the government. The diseases primarily involved pancreas and gastro-esophageal. Moreover, quality assessment tool in primary studies of diagnostic accuracy was mentioned in 58.3% (42/72) of the reviews, among which 88.1% (37/42) used the QUADAS tool, which met the demand of Cochrane Handbook 5.1, whereas STARD tool was rarely used.

**Table 1:** The characteristics of included SRs/MAs.

Items		n(%)
**Language of publications**	Chinese	7(9.7%)
	English	65(90.3%)
**Categories of disease**	Pancreas	23(31.9%)
	Gastro-esophageal	14(19.4%)
	Biliary	8(11.1%)
	Lung	6(8.3%)
	Colorectal	6(8.3%)
	Lymph nodes and Sarcoidosis	4(5.6%)
	Uterus	4(5.6%)
	Anus	3(4.2%)
	Prostate	1(1.4%)
**Category of journals**	SCI	63(87.5%)
**IF**	< 2	5(7.9%)
	2–5	34(54.0%)
	5–10	21(33.3%)
	>10	3(4.8%)
	Non-SCI	9(12.5%)
**Funding resources**	Fund	11(15.3%)
	business	0(0.0%)
	government	8(11.1%)
	institute / university	3(4.2%)
	professional organization	2(2.8%)
	Non-Fund	61(84.7%)
**Reporting conflict of interests**	Yes	33(45.8%)
	No	39(54.2%)
**Reporting of Keywords**	Yes	50(69.4%)
	No	22(30.6%)
**Application of quality assessment tool**	QUADAS	39(54.2%)
	STARD	5(6.9%)
	Non-reported	30(41.7%)
**Number of authors**	1–3	17(23.6%)
	4–9	52(72.2%)
	≥ 10	3(4.2%)

**Fig 2:**
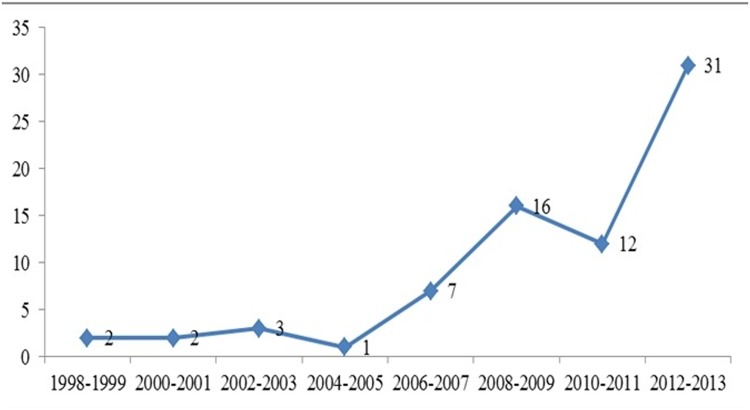
Publications of EUS diagnosis every two years.

**Fig 3:**
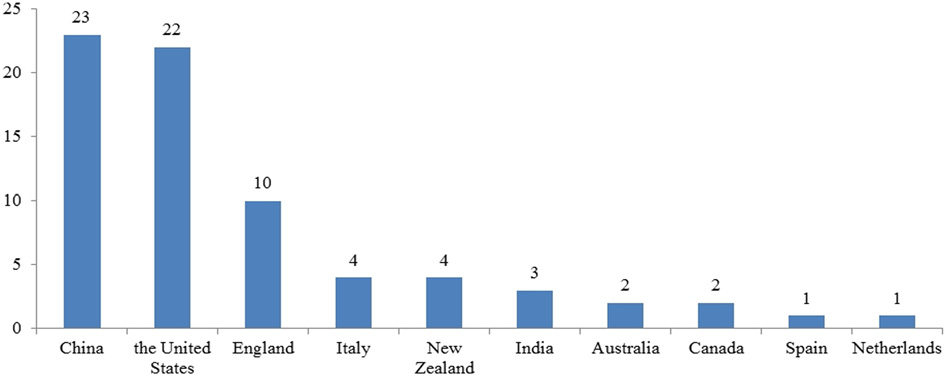
Number of the first author country in the field of EUS diagnosis.

As show in Fig 2, the number of publications on SRs/MAs of EUS diagnosis has been increasing over the last two decades. The first SR/MA was published in 1998, and 88.9% (64/72) articles went into publication after 2007. Fig 3 displays that publications with their authors coming from the United States and China play a prominent role in our study, but only 5 reviews are composed by cross-national authors.

### Reporting Quality

After assessing the compliance of papers with the 27-item PRISMA checklist, we got an overall score of 19.9±3.5, but not a single review met all the listing criteria in PRISMA statement, with its full details given in Table 2. For more than one-third items (10/27), 80.0% articles are in compliance with the criteria, but for the item of Rationale, all articles have met the criterion. On the contrary, there were 7 items whose compliance rates are below 50.0%, which were mainly considered in risk of bias and searching. Then, the quality of the remaining items was medium between 50% and 80% in compliance with PRISMA statement.

**Table 2:** PRISMA statement’s results [n(%)].

Items		Total(yes)	≥2009 vs ≤2008			SCI vs Non-SCI			Fund vs Non-Fund		
			≥2009(n = 49)	≤2008(n = 23)	p value	SCI (n = 63)	Non-SCI(n = 9)	p value	Fund(n = 11)	Non-Fund(n = 61)	p value
**Title**	1.Title	63(87.5%)	48(98.0%)	15(65.2%)	0.03	54(85.7%)	9(100.0%)	0.42	10(90.9%)	53(86.9%)	0.71
**Abstract**	2.Structured summary	65(90.3%)	47(96.0%)	18(78.3%)	0.03	56(88.9%)	9(100.0%)	0.54	10(90.9%)	55(90.2%)	0.94
**Introduction**	3.Rationale	72(100.0%)	49(100.0%)	23(100.0%)	—	63(100.0%)	9(100.0%)	—	11(100.0%)	61(100.0%)	—
**Methods**	4.Objective	66(91.7%)	44(89.8%)	22(95.7%)	0.42	62(98.4%)	4(44.4%)	0.0003	11(100.0%)	55(90.2%)	0.51
	5.Protocol and registration	0(0.0%)	0(0.0%)	0(0.0%)	—	0(0.0%)	0(0.0%)	—	0(0.0%)	0(0.0%)	—
	6.Eligibility criteria	37(51.4%)	28(57.1%)	9(39.1%)	0.16	32(50.8%)	5(55.6%)	0.79	5(45.5%)	32(52.5%)	0.67
	7.Information sources	61(84.7%)	44(89.8%)	17(73.9%)	0.09	56(88.9%)	5(55.6%)	0.02	10(90.9%)	51(83.6%)	0.54
	8.Search	8(11.1%)	7(14.3%)	1(4.4%)	0.24	7(11.1%)	1(11.1%)	1	1(9.1%)	7(11.5%)	0.82
	9.Study selection	50(69.4%)	37(75.5%)	13(56.5%)	0.11	46(73.0%)	4(44.4%)	0.09	7(63.6%)	43(70.5%)	0.65
	10.Data collection process	42(58.3%)	31(63.3%)	11(47.8%)	0.22	38(60.3%)	4(44.4%)	0.37	6(54.5%)	36(59.0%)	0.78
	11.Data items	43(59.7%)	32(65.3%)	11(47.8%)	0.16	39(61.9%)	4(44.4%)	0.32	9(81.8%)	34(55/7%)	0.12
	12.Risk of bias in individual studies	40(55.6%)	33(67.4%)	7(30.4%)	0.005	39(61.9%)	1(11.1%)	0.02	6(54.5%)	34(55/7%)	0.94
	13.Summary measures	69(95.8%)	47(95.9%)	22(95.7%)	0.96	61(96.8%)	8(88.9%)	0.3	11(100.0%)	58(95.1%)	0.84
	14.Synthesis of results	70(97.2%)	47(95.9%)	23(100.0%)	0.56	63(100.0%)	7(77.8%)	0.02	11(100.0%)	59(96.7%)	0.98
	15.Risk of bias across studies	36(50.0%)	28(57.1%)	8(34.8%)	0.08	33(52.4%)	3(33.3%)	0.29	6(54.5%)	30(49.2%)	0.74
	16.Additional analyses	22(30.6%)	18(36.7%)	4(17.4%)	0.1	18(28.6%)	4(44.4%)	0.34	2(18.2%)	20(32.8%)	0.34
**Results**	17.Study selection	52(72.2%)	39(79.6%)	12(52.2%)	0.02	50(79.4%)	1(11.1%)	0.002	8(72.7%)	43(70.5%)	0.88
	18.Study characteristics	63(87.5%)	45(91.8%)	18(78.3%)	0.12	56(88.9%)	7(77.8%)	0.36	9(81.8%)	54(88.5%)	0.54
	19.Risk of bias with studies	34(47.2%)	24(49.0%)	10(43.5%)	0.66	33(52.4%)	1(11.1%)	0.05	5(45.5%)	29(47.5%)	0.9
	20.Results of individual studies	71(98.6%)	48(98.0%)	23(100.0%)	0.82	63(100.0%)	8(88.9%)	0.06	11(100.0%)	60(98.4%)	0.74
	21.Synthesis of results	71(98.6%)	48(98.0%)	23(100.0%)	0.82	62(98.4%)	9(100.0%)	0.64	11(100.0%)	60(98.4%)	0.74
	22.Risk of bias across studies	32(44.4%)	26(53.1%)	6(26.1%)	0.04	29(46.0%)	3(33.3%)	0.48	6(54.5%)	26(42.6%)	0.47
	23.Additional analyses	34(47.2%)	26(53.1%)	8(34.8%)	0.15	30(47.62%)	4(44.4%)	0.86	4(36.4%)	30(49.2%)	0.44
**Discussion**	24.Summary of evidence	72(100.0%)	49(100.0%)	23(100.0%)	—	63(100.0%)	9(100.0%)	—	11(100.0%)	61(100.0%)	—
	25.Limitations	38(52.8%)	29(59.2%)	9(39.1%)	0.12	34(54.0%)	4(44.4%)	0.59	8(72.7%)	30(49.2%)	0.16
	26.Conclusions	68(94.4%)	45(91.8%)	23(100.0%)	0.31	62(98.4%)	6(66.7%)	0.005	11(100.0%)	57(93.4%)	0.7
**Funding**	27.Funding	16(22.2%)	12(24.5%)	4(17.4%)	0.5	15(23.8%)	1(11.1%)	0.41	10(90.9%)	6(9.8%)	<0.0001

**Fig 4:**
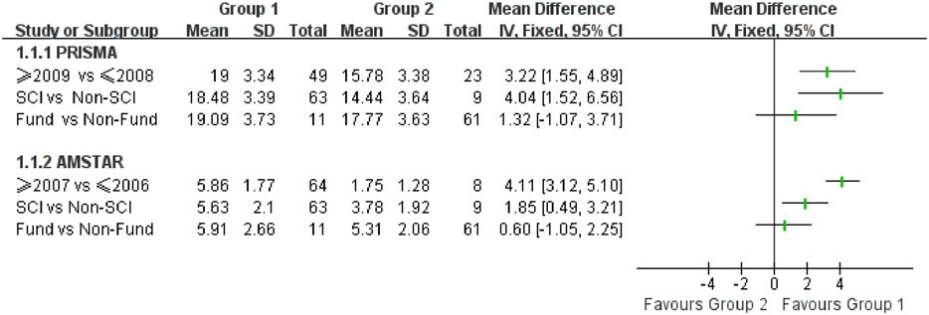
Forest plot for subgroup analysis on PRISMA and AMSTAR statements.

As no CSRs were obtained in this study, category of reviews (CSR vs. non-CSR) could not be analyzed. Thus, analysis for the other three potential factors was performed carefully and the result is shown in Table 2. It can be seen that 68.0% (49/72) reviews were published after 2009. Between the two periods (≥2009 vs. ≤2008), difference is statistically significant in a number of items, including title, structured summary, risk of bias in individual in the method part, study selection and risk of bias across studies in the result part. However, no difference was found in rationale, protocol, and summary of evidence after the release of PRISMA. Apart from Table 2, the forest plot (Fig 4) also clearly displays the difference with a mean of 3.22 (95% CI: 1.59 to 4.89). In addition, Table 2 and Fig 4 jointly indicate that SRs/MAs from SCI are of higher quality than non-SCI studies. To be specific, there exists a significant difference in items such as risk of bias in individual, study selection and conclusions. Yet there was no evidence suggesting a difference in the last item “funding of studies”.

### Methodological Quality

Compliance with the AMSTAR checklist, the score of all SRs/MAs was mere 5.4±2.2. Most articles (88.9%, 64/72) were published after 2007, the release time of AMSTAR. Table 3 displays that the compliance rate with a certain item in the checklist ranges from 1.4% to 90.3%, and nearly half (5/11) of them are beyond 50%. The optimum item was “characteristic of the included studies” the same as PRISMA. However, there were still 6 items with their compliance rates less than 50%. The worst condition occurs in the items of “preliminary design” (1.4%) and “comprehensive literature search” (11.1%).

**Table 3:** AMSTAR tool’s results [n(%)].

Item	Total(yes)	≥2007 vs ≤2006			SCI vs Non-SCI			Fund vs Non-Fund		
		≥2007(n = 64)	≤2006(n = 8)	p value	SCI (n = 63)	Non-SCI(n = 9)	p value	Fund(n = 11)	Non-Fund(n = 61)	p value
1. Was an ‘a priori’ design provided?	1(1.4%)	1(1.6%)	0(0.0%)	0.59	1(1.8%)	0(0.0%)	0.64	0(0.0%)	1(1.6%)	0.74
2. Was there duplicate study selection and data extraction?	38(52.8%)	37(57.8%)	1(12.5%)	0.04	29(50.9%)	9(60.0%)	0.22	6(54.5%)	32(52.5%)	0.9
3. Was a comprehensive literature search performed?	8(11.1%)	8(12.5%)	0(0.0%)	0.53	7(12.3%)	1(6.7%)	1.00	1(9.1%)	7(11.5%)	0.82
4. Was the status of publication used as an inclusion criterion?	29(40.3%)	28(43.8%)	1(12.5%)	0.12	24(42.1%)	5(33.3%)	0.09	5(45.5%)	24(39.3%)	0.7
5. Was a list of studies provided?	45(62.5%)	45(70.3%)	0(0.0%)	0.01	39(68.4%)	6(40.05)	0.01	8(72.7%)	37(60.7%)	0.45
6. Were the characteristics of the included studies provided?	65(90.3%)	61(95.3%)	4(50.0%)	0.001	50(87.7%)	15(100.0%)	0.54	9(81.8%)	56(91.8%)	0.32
7. Was the scientific quality of the included studies assessed and documented?	35(48.6%)	34(53.1%)	1(12.5%)	0.06	29(50.9%)	6(40.0%)	0.79	6(54.5%)	29(47.5%)	0.67
8. Was the scientific quality of the included studies used appropriately in formulating conclusion?	56(77.8%)	51(79.7%)	5(62.5%)	0.28	48(84.2%)	8(53.3%)	0.40	9(81.8%)	47(77.0%)	0.73
9. Were the methods used to combine the findings of studies appropriate?	50(69.4%)	49(76.6%)	1(12.5%)	0.005	42(73.7%)	8(53.3%)	0.85	9(81.8%)	41(67.2%)	0.34
10. Was the likelihood of publication bias assessed?	31(43.1%)	31(48.4%)	0(0.0%)	0.06	23(40.4%)	8(53.3%)	0.53	6(54.5%)	25(41.0%)	0.41
11. Was the conflict of interest stated?	31(43.1%)	30(46.9%)	1(12.5%)	0.1	30(52.6%)	1(6.7%)	0.07	6(54.5%)	25(41.0%)	0.41

After 2007, significant difference was found in four items including study selection and data extraction, eligibility criterion, characteristics of the included studies, and statistical methods. Generally speaking, the overall quality of articles from SCI was better than that of non-SCI papers, especially in the item of “a list of studies provided”. Fig 4 reveals a great improvement with a mean of 4.11 (95% CI: 3.12 to 5.10) after AMSTAR’s release. Similarly, funding resource had no influence on methodological quality.

### Multiple linear regression analysis

To assess the independent contributions of variables in affecting the quality of reporting or methodology, two multiple linear regression models were established with backward selection procedure (α_in_ = 0.05,α_out_ = 0.1, model 1-PRISMA, model 2-AMSTAR) in Table 4. After assessing all potential factors by SPSS 17.0 software, funding resource is excluded from models apparently, while the other two factors are retained (*p*<0.05) in both models.

**Table 4:** Estimate of the effects of multiple linear regression analysis.

Model		Non-Standardized regression coefficients		Standardized regression coefficients	T value	P value	β_m_ 95%CI	
		β_m_	SE				lower	upper
**1**	Constant	13.797	1.162		11.879	0	11.48	16.12
	Publication time	3.915	0.738	0.524	5.308	0	2.444	5.387
	SCI	3.916	1.04	0.372	3.766	0	1.842	5.991
**2**	Constant	1.279	0.656		1.95	0.055	-0.029	2.587
	Publication time	2.811	0.416	0.613	6.75	0	1.98	3.642
	SCI	2.526	0.587	0.391	4.303	0	1.355	3.698

β_m_: Partial regression coefficient

After controlling the factor of SCI in model 1, the final score got improved to 3.915 by publication time (95%CI: 2.444, 5.387). All other results are presented clearly in Table 4. In both models, the total score of quality assessment was greatly involved with time of publication and inclusion in the SCI. Moreover, it indicates that the standardized regression coefficient of publication time is higher than that of SCI (0.524>0.372 in model 1, 0.613>0.391 in model 2).

## Discussion

### Summary of evidence

In general, the number of SRs/MAs of EUS diagnosis is increasing annually. EUS is widely applied in the diagnosis of digestive system disease. The majority of SRs/MAs have been conducted in the United States and China. Although cross-national research is becoming common, only 5 of the assessed reviews of EUS diagnosis were cross-national. Of the 72 SRs/MAs assessed, 57 were from the SCI and 11 had funding resources. This study showed that the reporting and methodology of EUS diagnosis was of average quality, with overall average scores on the PRISMA and AMSTAR criteria of 19.9 and 5.4, respectively. Subgroup analysis for time of publication showed a substantial improvement in certain aspects of adherence after the publication of generic criteria, but there is still considerable room for improvement in some criteria, especially protocol and searching, which require urgent measures in order to improve.

One conclusion we can draw is that less attention has been paid to protocol and registration, because none of the reviews reported on this criterion, and this was true of reviews across the time period sampled. There were various reasons why researchers refused to be registered. Silagy’s (2002) report compared CSRs with their previously published protocols. Over 90% of studies showed major changes, revealing the limitations of protocol and registration[20,21]. However, measures have since been taken to improve this process, such as reporting the reason for changing especially the specified outcomes[22], and it is clear that publication of protocols adds scientific credibility and improves research standards[23]. Simultaneously, for non-CSR research, the PROSPERO registration platform was created to help researchers control publication and reporting bias, and to reduce unplanned duplication of SRs/MAs[24,25]. These measures may help to enhance the development of high-quality SRs.

Both PRISMA and AMSTAR statements mention the importance of a literature search, but our findings showed that compliance with these items was very poor (below 20%). Incomplete search strategies may produce reporting bias and lead researchers or clinicians to misinterpret the reliability of the evidence [8,9]. Since the lack of a literature search can restrict the overall quality of a study, future authors should focus on ways to refine the searching process and pay more attention to the following requirements: 1) search by two authors, 2) search at least two electronic databases as well as hand searching, 3) seek grey literature and recount references, 4) not restrict language, and 5) report the literature search process [26,27]. Although it is difficult to establish a robust searching strategy, this is an area that needs to show improvement in future studies. In addition, only half of SRs/MAs described eligibility criteria in detail. This information needs to be made more apparent and presented much more clearly by use of appropriate tables and figures.

Through subgroup analysis and regression analysis for factors that may affect the quality of the research, we concluded that time of publication and SCI relate more to the overall quality of SRs/MAs than does funding resource. The introduction of PRISMA and AMSTAR statements has largely improved the quality of SRs/MAs. The role of the SCI as a potential factor in the quality of the research suggests that this should be a requirement for future reviews. Only one-fifth of journals require reporting strictly according to the PRISMA statement and this seems to be irrelevant to IF. Since this proportion is so low, the PRISMA statement should feature more in journal instructions to authors, especially for non-SCI journals. Although each set of reporting standards has distinctive advantages, understanding and dissemination of a generic assessment tool was still inadequate. The task of collating information in order to produce guidance on criteria that present researchers with problems will continue [28]. Although the Cochrane Database of Systematic Reviews (CDSR) is rigorously managed and has a robust registration platform [20], we could not distinguish CSR from non-CSR articles because the full text of CSRs was not currently available. It is hoped that research in this field will have a chance to appear on the CDSR.

In addition to including the PRISMA statement in journal instructions to authors, reviewers need to pay more attention to those items that our research identified as having a low reporting rate, such as literature searching. For authors and researchers, using PRISMA and AMSTAR is an effective way to design research in the primary stage, and to test its effectiveness in the final stage. Understanding and mastering relevant procedures is essential for criteria to be successfully met. Future methodological researchers need to focus more on literature searching and seek more guidance concerning criteria that they find problematic.

According to the Cochrane Handbook 5.1, the QUADAS tool is regarded as a common tool to assess the quality of primary diagnostic research [29]. The superiority of the revised tool (QUADAS-2) was apparent in the quality assessment of diagnostic accuracy studies [30,31]. However, nearly half of SRs/MAs did not report using any quality assessment tool. This low proportion may be because QUADAS is not as popular as we thought. Future researchers should be encouraged to apply QUADAS-2.

### Strengths and Limitations

Although we assessed the SRs/MAs strictly according to the standardized statements, the results of this evaluation were not without limitations. First, four English databases and three Chinese databases were searched, instead of a specific professional journal or a non-English and Chinese database. Second, a further potential limitation was publication bias. There were several SRs/MAs from the same author, such as *Puli*, *Srinivas R*, who published ten articles of the articles we studied. Third, although two reviewers had received rigorous training before undertaking quality assessment, their differing understanding of the two standards is a potential limitation. Last, the weight of the measured item on these two statements may be different, but we regarded each item as the same weight.

Despite these limitations, our research clearly evaluated the need for improving quality for reviews/analyses of a diagnostic approach. First, this was the first article that used two kinds of independent standards to evaluate reporting and methodological quality in global diagnosis SRs/MAs. Second, the findings could be important in the field of EUS diagnosis when clinicians need to refer to SRs/MAs to judge the effectiveness of EUS diagnosis. Third, this study was carefully designed and searched seven main databases. Finally, it is the first, to our knowledge, to analyze potential factors (publication time, SCI, and funding) affecting the quality of SRs/MAs. Our findings could guide future research in this diagnostic field.

## Conclusion

With the introduction of PRISMA and AMSTAR statements, the reporting and methodological quality of SRs/MAs of EUS diagnosis has improved measurably over the last two decades. It is hoped that research in this field will have a chance to appear on CDSR. Similarly, literature searching and protocol and registration criteria need to be addressed more in the future. Time of publication and SCI relate more to the overall quality of SRs/MAs than does funding resource. Future researchers should be encouraged to apply QUADAS-2.

## Supporting Information

S1 Text Search algorithms. (DOC)

S2 Text References to systematic reviews in this review. (DOCX)
