# Supplementary material for: Quality Assessment and Factor Analysis of Systematic Reviews and Meta-Analyses of Endoscopic Ultrasound Diagnosis
Source: PLoS One. 2015 Apr 23;10(4):e0120911. doi: 10.1371/journal.pone.0120911 (PMC4408104; doi:10.1371/journal.pone.0120911)
Supplement: S1 Text — (DOC) [file pone.0120911.s001.doc]

The syntax for **PubMed** searches was as follows:

#1 "endosonography"[MeSH Terms]

#2 " endosonography "[Title/Abstract]

#3 "endoscopic ultrasound"[Title/Abstract]

#4 "ultrasongraphy"[Title/Abstract]

#5 "endoscopic echography"[Title/Abstract]

#6 #1 OR #2 OR #3OR #4 OR #5

#7 "systematic review*[Title/Abstract] OR meta analysis[Title/Abstract] OR meta-analyses[Title/Abstract] OR meta-analysis[Title/Abstract] OR meta analyses[Title/Abstract]

#8 #6 OR #7

The syntax for **EMBASE** searches was as follows:

#1 "endoscopic echography" [MeSH Terms]

#2 '"endoscopic echography":ab,ti

#3 "endoscopic ultrasound":ab,ti

#4 "ultrasongraphy":ab,ti

#5 " endosonography ":ab,ti

#6 #1 OR #2 OR #3OR #4 OR #5

#7"systematic review*:ab,ti OR meta analysis:ab,ti OR meta-analyses:ab,ti OR meta-analysis:ab,ti OR "meta analyses":ab,ti

#8 #6AND #7

#9 #6AND #7 AND [medline]/lim

#10 #8 NOT #9

The syntax for **The** **Cochrane Library** searches was as follows:

#1 " endosonography ":ti,ab,kw

#2 " endoscopic ultrasonography ":ti,ab,kw endoscopic ultrasound

#3 " endoscopic ultrasound ":ti,ab,kw

#4 '"ultrasongraphy":ti,ab,kw

#5'"endoscopic echography": ti,ab,kw

#6 "EUS":ti,ab,kw

#7 "endosnography":MeSH descriptor

#8 #1 OR #2 OR #3OR #4 OR #5 OR #6 OR #7

The syntax for **Web of science** searches was as follows:

Topic=(systematic review* OR meta analyses OR meta-analyses OR meta-analysis OR meta analyses OR meta analysis) AND Topic=(endosonography OR endoscopic ultrasonography OR endoscopic ultrasound OR ultrasongraphy OR endoscopic echography)
