# Supplementary material for: Quality Assessment and Factor Analysis of Systematic Reviews and Meta-Analyses of Endoscopic Ultrasound Diagnosis
Source: PLoS One. 2015 Apr 23;10(4):e0120911. doi: 10.1371/journal.pone.0120911 (PMC4408104; doi:10.1371/journal.pone.0120911)
Supplement: S2 Text — (DOCX) [file pone.0120911.s002.docx]

**REFERENCES**

**References to systematic reviews in this review**

1. Li X, Xu W, Shi J, [Lin Y](http://www.ncbi.nlm.nih.gov/pubmed?term=Lin%20Y%5BAuthor%5D&cauthor=true&cauthor_uid=24115828), [Zeng X](http://www.ncbi.nlm.nih.gov/pubmed?term=Zeng%20X%5BAuthor%5D&cauthor=true&cauthor_uid=24115828). Endoscopic ultrasound elastography for differentiating between pancreatic adenocarcinoma and inflammatorymasses: a meta-analysis. World J Gastroenterol. 2013;19:6284-91.

2. Cardoso R, Coburn N, Seevaratnam R,  [Sutradhar R](http://www.ncbi.nlm.nih.gov/pubmed?term=Sutradhar%20R%5BAuthor%5D&cauthor=true&cauthor_uid=22237654), [Lourenco LG](http://www.ncbi.nlm.nih.gov/pubmed?term=Lourenco%20LG%5BAuthor%5D&cauthor=true&cauthor_uid=22237654), [Mahar A](http://www.ncbi.nlm.nih.gov/pubmed?term=Mahar%20A%5BAuthor%5D&cauthor=true&cauthor_uid=22237654), et al. A systematic review and meta-analysis of the utility of EUS for preoperative staging for gastric cancer. Gastric Cancer. 2012;15:S19-26.

3. Hewitt MJ, McPhail MJ, Possamai L,  [Dhar A](http://www.ncbi.nlm.nih.gov/pubmed?term=Dhar%20A%5BAuthor%5D&cauthor=true&cauthor_uid=22248600), [Vlavianos P](http://www.ncbi.nlm.nih.gov/pubmed?term=Vlavianos%20P%5BAuthor%5D&cauthor=true&cauthor_uid=22248600), [Monahan KJ](http://www.ncbi.nlm.nih.gov/pubmed?term=Monahan%20KJ%5BAuthor%5D&cauthor=true&cauthor_uid=22248600).. EUS-guided FNA for diagnosis of solid pancreatic neoplasms: a meta-analysis. Gastrointest Endosc. 2012;75:319-31.

4. Adams K, Shah PL, Edmonds L, Lim E. Test performance of endobronchial ultrasound and transbronchial needle aspiration biopsy for mediastinal staging in patients with lung cancer: systematic review and meta-analysis. Thorax. 2009;64:757-62.

5. Gu P, Zhao YZ, Jiang LY,  Zhang W, Xin Y, Han BH. Endobronchial ultrasound-guided transbronchial needle aspiration for staging of lung cancer: a systematic review and meta-analysis. Eur J Cancer. 2009;45:1389-96.

6. Gong TT, Hu DM, Zhu Q. Contrast-enhanced EUS for differential diagnosis of pancreatic mass lesions: a meta-analysis. Gastrointest Endosc. 2012;76:301-9.

7. Hébert-Magee S, Bae S, Varadarajulu S, [Frost AR](http://www.ncbi.nlm.nih.gov/pubmed?term=Frost%20AR%5BAuthor%5D&cauthor=true&cauthor_uid=23711182), [Eloubeidi MA](http://www.ncbi.nlm.nih.gov/pubmed?term=Eloubeidi%20MA%5BAuthor%5D&cauthor=true&cauthor_uid=23711182), [Eltoum IA](http://www.ncbi.nlm.nih.gov/pubmed?term=Eltoum%20IA%5BAuthor%5D&cauthor=true&cauthor_uid=23711182). The presence of a cytopathologist increases the diagnostic accuracy of endoscopic ultrasound-guided fine needle aspiration cytology for pancreatic adenocarcinoma: a meta-analysis. Cytopathology. 2013;24:159-71.

8. Tse F, Liu L, Barkun AN, Armstrong D, Moayyedi P. EUS: a meta-analysis of test performance in suspected choledocholithiasis. Gastrointest Endosc 2008;67:235-44.

9. Zhang R, Ying K, Shi L, Zhang L, Zhou L. Combined endobronchial and endoscopic ultrasound-guided fine needle aspiration for mediastinal lymph node staging of lung cancer: a meta-analysis. Eur J Cancer. 2013;49:1860-7.

10. Puli SR, Reddy JB, Bechtold ML, [Choudhary A](http://www.ncbi.nlm.nih.gov/pubmed?term=Choudhary%20A%5BAuthor%5D&cauthor=true&cauthor_uid=19219506), [Antillon MR](http://www.ncbi.nlm.nih.gov/pubmed?term=Antillon%20MR%5BAuthor%5D&cauthor=true&cauthor_uid=19219506), [Brugge WR](http://www.ncbi.nlm.nih.gov/pubmed?term=Brugge%20WR%5BAuthor%5D&cauthor=true&cauthor_uid=19219506). Accuracy of endoscopic ultrasound to diagnose nodal invasion by rectal cancers: a meta-analysis and systematic review. Ann Surg Oncol. 2009;16:1255-65.

11. Xu W, Shi J, Zeng X,  Li X, Xie WF, Guo J, et al. EUS elastography for the differentiation of benign and malignant lymph nodes: a meta-analysis. Gastrointest Endosc. 2011;74:1001-9

12. Puli SR, Batapati Krishna Reddy J, Bechtold ML, Antillon MR, Ibdah JA. How good is endoscopic ultrasound for TNM staging of gastric cancers? A meta-analysis and systematic review. World J Gastroenterol. 2008;14:4011-9.

13. Puli SR, Batapati Krishna Reddy J, Bechtold ML,  [Ibdah JA](http://www.ncbi.nlm.nih.gov/pubmed?term=Ibdah%20JA%5BAuthor%5D&cauthor=true&cauthor_uid=18494054), [Antillon D](http://www.ncbi.nlm.nih.gov/pubmed?term=Antillon%20D%5BAuthor%5D&cauthor=true&cauthor_uid=18494054), [Singh S](http://www.ncbi.nlm.nih.gov/pubmed?term=Singh%20S%5BAuthor%5D&cauthor=true&cauthor_uid=18494054). Endoscopic ultrasound: it's accuracy in evaluating mediastinal lymphadenopathy? A meta-analysis and systematic review. World J Gastroenterol. 2008;14:3028-37.

14. Puli SR, Reddy JB, Bechtold ML,  Antillon D, Ibdah JA, Antillon MR. Staging accuracy of esophageal cancer by endoscopic ultrasound: a meta-analysis and systematic review. World J Gastroenterol. 2008;14:1479-90.

15. Puli SR, Bechtold ML, Reddy JB, [Choudhary A](http://www.ncbi.nlm.nih.gov/pubmed?term=Choudhary%20A%5BAuthor%5D&cauthor=true&cauthor_uid=19018597), [Antillon MR](http://www.ncbi.nlm.nih.gov/pubmed?term=Antillon%20MR%5BAuthor%5D&cauthor=true&cauthor_uid=19018597), [Brugge WR](http://www.ncbi.nlm.nih.gov/pubmed?term=Brugge%20WR%5BAuthor%5D&cauthor=true&cauthor_uid=19018597). How good is endoscopic ultrasound in differentiating various T stages of rectal cancer? Meta-analysis andsystematic review. Ann Surg Oncol. 2009;16:254-65.

16. Chen J, Yang R, Lu Y, , [Xia Y](http://www.ncbi.nlm.nih.gov/pubmed?term=Xia%20Y%5BAuthor%5D&cauthor=true&cauthor_uid=22752601), [Zhou H](http://www.ncbi.nlm.nih.gov/pubmed?term=Zhou%20H%5BAuthor%5D&cauthor=true&cauthor_uid=22752601). Diagnostic accuracy of endoscopic ultrasound-guided fine-needle aspiration for solid pancreatic lesion: a systematic review. J Cancer Res Clin Oncol. 2012;138:1433-41.

17. Mei M, Ni J, Liu D, Jin P, Sun L. EUS elastography for diagnosis of solid pancreatic masses: a meta-analysis. Gastrointest Endosc. 2013;77:578-89.

18. Mocellin S, Marchet A, Nitti D. EUS for the staging of gastric cancer: a meta-analysis. Gastrointest Endosc. 2011;73:1122-34.

19. Wang KX, Ben QW, Jin ZD, Du YQ, Zou DW, Liao Z, et al. Assessment of morbidity and mortality associated with EUS-guided FNA: a systematic review. Gastrointest Endosc. 2011;73:283-90.

20. Puli SR, Singh S, Hagedorn CH, [Reddy J](http://www.ncbi.nlm.nih.gov/pubmed?term=Reddy%20J%5BAuthor%5D&cauthor=true&cauthor_uid=17350008), [Olyaee M](http://www.ncbi.nlm.nih.gov/pubmed?term=Olyaee%20M%5BAuthor%5D&cauthor=true&cauthor_uid=17350008). Diagnostic accuracy of EUS for vascular invasion in pancreatic and periampullary cancers: a meta-analysis and systematic review. Gastrointest Endosc. 2007;65:788-97.

21. Puli SR, Reddy JB, Bechtold ML, Antillon MR, Ibdah JA. Accuracy of endoscopic ultrasound in the diagnosis of distal and celiac axis lymph node metastasis inesophageal cancer: a meta-analysis and systematic review. Dig Dis Sci. 2008;53:2405-14.

22. Schmidt RL, Witt BL, Matynia AP, Barraza G, Layfield LJ, Adler DG. Rapid on-site evaluation increases endoscopic ultrasound-guided fine-needle aspiration adequacy for pancreatic lesions. Dig Dis Sci. 2013;58:872-82.

23. Affolter KE, Schmidt RL, Matynia AP,  [Adler DG](http://www.ncbi.nlm.nih.gov/pubmed?term=Adler%20DG%5BAuthor%5D&cauthor=true&cauthor_uid=23086117), [Factor RE](http://www.ncbi.nlm.nih.gov/pubmed?term=Factor%20RE%5BAuthor%5D&cauthor=true&cauthor_uid=23086117). Needle size has only a limited effect on outcomes in EUS-guided fine needle aspiration: a systematic review and meta-analysis. Dig Dis Sci. 2013;58:1026-34.

24. Puli SR, Bechtold ML, Reddy JB, [Choudhary A](http://www.ncbi.nlm.nih.gov/pubmed?term=Choudhary%20A%5BAuthor%5D&cauthor=true&cauthor_uid=19517233), [Antillon MR](http://www.ncbi.nlm.nih.gov/pubmed?term=Antillon%20MR%5BAuthor%5D&cauthor=true&cauthor_uid=19517233). Can endoscopic ultrasound predict early rectal cancers that can be resected endoscopically? A meta-analysisand systematic review. Dig Dis Sci. 2010;55:1221-9.

25. Agarwal R, Srinivasan A, Aggarwal AN, Gupta D. Efficacy and safety of convex probe EBUS-TBNA in sarcoidosis: a systematic review and meta-analysis. Respir Med. 2012;106:883-92.

26. Wang Z, Chen JQ. Imaging in assessing hepatic and peritoneal metastases of gastric cancer: a systematic review. BMC Gastroenterol. 2011;11:19.

27. Kwee RM, Kwee TC. Imaging in local staging of gastric cancer: a systematic review. J Clin Oncol. 2007;25:2107-16.

28. Chandra S, Nehra M, Agarwal D, Mohan A. Diagnostic accuracy of endobronchial ultrasound-guided transbronchial needle biopsy in mediastinal lymph adenopathy: a systematic review and meta-analysis. Respir Care. 2012;57:384-91.

29. Micames CG, McCrory DC, Pavey DA,  Jowell PS, Gress FG. Endoscopic ultrasound-guided fine-needle aspiration for non-small cell lung cancer staging: A systematic review and meta analysis. Chest. 2007;131:539-48.

30. Verma D, Kapadia A, Eisen GM, Adler DG. EUS vs MRCP for detection of choledocholithiasis. Gastrointest Endosc. 2006;64:248-54.

31. Worrell S, Horvath K, Blakemore T, Flum D. Endorectal ultrasound detection of focal carcinoma within rectal adenomas. Am J Surg. 2004;187:625-9

32. Kelly S, Harris KM, Berry E, [Hutton J](http://www.ncbi.nlm.nih.gov/pubmed?term=Hutton%20J%5BAuthor%5D&cauthor=true&cauthor_uid=11559651), [Roderick P](http://www.ncbi.nlm.nih.gov/pubmed?term=Roderick%20P%5BAuthor%5D&cauthor=true&cauthor_uid=11559651), [Cullingworth J](http://www.ncbi.nlm.nih.gov/pubmed?term=Cullingworth%20J%5BAuthor%5D&cauthor=true&cauthor_uid=11559651), et al. A systematic review of the staging performance of endoscopic ultrasound in gastro-oesophageal carcinoma. Gut. 2001;49:534-9.

33. van Vliet EP, Heijenbrok-Kal MH, Hunink MG, [Kuipers EJ](http://www.ncbi.nlm.nih.gov/pubmed?term=Kuipers%20EJ%5BAuthor%5D&cauthor=true&cauthor_uid=18212745), [Siersema PD](http://www.ncbi.nlm.nih.gov/pubmed?term=Siersema%20PD%5BAuthor%5D&cauthor=true&cauthor_uid=18212745). Staging investigations for oesophageal cancer: a meta-analysis. Br J Cancer. 2008;98:547-57.

34. Thosani N, Singh H, Kapadia A, [Ochi N](http://www.ncbi.nlm.nih.gov/pubmed?term=Ochi%20N%5BAuthor%5D&cauthor=true&cauthor_uid=22115605), [Lee JH](http://www.ncbi.nlm.nih.gov/pubmed?term=Lee%20JH%5BAuthor%5D&cauthor=true&cauthor_uid=22115605), [Ajani J](http://www.ncbi.nlm.nih.gov/pubmed?term=Ajani%20J%5BAuthor%5D&cauthor=true&cauthor_uid=22115605), et al. Diagnostic accuracy of EUS in differentiating mucosal versus submucosal invasion of superficial esophagealcancers: a systematic review and meta-analysis. Gastrointest Endosc. 2012;75:242-53.

35. Thosani N, Thosani S, Qiao W, [Fleming JB](http://www.ncbi.nlm.nih.gov/pubmed?term=Fleming%20JB%5BAuthor%5D&cauthor=true&cauthor_uid=20694512), [Bhutani MS](http://www.ncbi.nlm.nih.gov/pubmed?term=Bhutani%20MS%5BAuthor%5D&cauthor=true&cauthor_uid=20694512), [Guha S](http://www.ncbi.nlm.nih.gov/pubmed?term=Guha%20S%5BAuthor%5D&cauthor=true&cauthor_uid=20694512). Role of EUS-FNA-based cytology in the diagnosis of mucinous pancreatic cystic lesions: a systematic review and meta-analysis. Dig Dis Sci. 2010;55:2756-66.

36. Ying L, Lin X, Xie ZL, [Hu YP](http://www.ncbi.nlm.nih.gov/pubmed?term=Hu%20YP%5BAuthor%5D&cauthor=true&cauthor_uid=23731128), [Tang KF](http://www.ncbi.nlm.nih.gov/pubmed?term=Tang%20KF%5BAuthor%5D&cauthor=true&cauthor_uid=23731128), [Shi KQ](http://www.ncbi.nlm.nih.gov/pubmed?term=Shi%20KQ%5BAuthor%5D&cauthor=true&cauthor_uid=23731128). Clinical utility of endoscopic ultrasound elastography for identification of malignant pancreatic masses: a meta-analysis. J Gastroenterol Hepatol. 2013;28:1434-43.

37. Sajid MS, Khatri K, Siddiqui MR, Baig MK. Endo-anal ultrasound versus endo-anal magnetic resonance imaging for the depiction of external anal sphincterpathology in patients with faecal incontinence: a systematic review. Magy Seb. 2010;63:9-15.

38. Siddiqui MR, Ashrafian H, Tozer P, [Daulatzai N](http://www.ncbi.nlm.nih.gov/pubmed?term=Daulatzai%20N%5BAuthor%5D&cauthor=true&cauthor_uid=22513437), [Burling D](http://www.ncbi.nlm.nih.gov/pubmed?term=Burling%20D%5BAuthor%5D&cauthor=true&cauthor_uid=22513437), [Hart A](http://www.ncbi.nlm.nih.gov/pubmed?term=Hart%20A%5BAuthor%5D&cauthor=true&cauthor_uid=22513437), et al. A diagnostic accuracy meta-analysis of endoanal ultrasound and MRI for perianal fistula assessment. Dis Colon Rectum. 2012;55:576-85.

39. Tan E, Anstee A, Koh DM, [Gedroyc W](http://www.ncbi.nlm.nih.gov/pubmed?term=Gedroyc%20W%5BAuthor%5D&cauthor=true&cauthor_uid=18330582), [Tekkis PP](http://www.ncbi.nlm.nih.gov/pubmed?term=Tekkis%20PP%5BAuthor%5D&cauthor=true&cauthor_uid=18330582). Diagnostic precision of endoanal MRI in the detection of anal sphincter pathology: a meta-analysis. Int J Colorectal Dis. 2008;23:641-51.

40. Dewitt J, Devereaux BM, Lehman GA, [Sherman S](http://www.ncbi.nlm.nih.gov/pubmed?term=Sherman%20S%5BAuthor%5D&cauthor=true&cauthor_uid=16675307), [Imperiale TF](http://www.ncbi.nlm.nih.gov/pubmed?term=Imperiale%20TF%5BAuthor%5D&cauthor=true&cauthor_uid=16675307). Comparison of endoscopic ultrasound and computed tomography for the preoperative evaluation of pancreatic cancer: a systematic review. Clin Gastroenterol Hepatol. 2006;4:717-25.

41. Chen G, Liu S, Zhao Y, [Dai M](http://www.ncbi.nlm.nih.gov/pubmed?term=Dai%20M%5BAuthor%5D&cauthor=true&cauthor_uid=23719604), [Zhang T](http://www.ncbi.nlm.nih.gov/pubmed?term=Zhang%20T%5BAuthor%5D&cauthor=true&cauthor_uid=23719604). Diagnostic accuracy of endoscopic ultrasound-guided fine-needle aspiration for pancreatic cancer: a meta-analysis. Pancreatology. 2013;13:298-304.

42. Thornton GD, McPhail MJ, Nayagam S, [Hewitt MJ](http://www.ncbi.nlm.nih.gov/pubmed?term=Hewitt%20MJ%5BAuthor%5D&cauthor=true&cauthor_uid=23395570), [Vlavianos P](http://www.ncbi.nlm.nih.gov/pubmed?term=Vlavianos%20P%5BAuthor%5D&cauthor=true&cauthor_uid=23395570), [Monahan KJ](http://www.ncbi.nlm.nih.gov/pubmed?term=Monahan%20KJ%5BAuthor%5D&cauthor=true&cauthor_uid=23395570). Endoscopic ultrasound guided fine needle aspiration for the diagnosis of pancreatic cystic neoplasms: a meta-analysis. Pancreatology. 2013;13:48-57.

43. Tang S, Huang G, Liu J, [Liu T](http://www.ncbi.nlm.nih.gov/pubmed?term=Liu%20T%5BAuthor%5D&cauthor=true&cauthor_uid=19854016), [Treven L](http://www.ncbi.nlm.nih.gov/pubmed?term=Treven%20L%5BAuthor%5D&cauthor=true&cauthor_uid=19854016), [Song S](http://www.ncbi.nlm.nih.gov/pubmed?term=Song%20S%5BAuthor%5D&cauthor=true&cauthor_uid=19854016), et al. Usefulness of 18F-FDG PET, combined FDG-PET/CT and EUS in diagnosing primary pancreatic carcinoma: a meta-analysis. Eur J Radiol. 2011;78:142-50.

44. Pei Q, Zou X, Zhang X, [Chen M](http://www.ncbi.nlm.nih.gov/pubmed?term=Chen%20M%5BAuthor%5D&cauthor=true&cauthor_uid=23127527), [Guo Y](http://www.ncbi.nlm.nih.gov/pubmed?term=Guo%20Y%5BAuthor%5D&cauthor=true&cauthor_uid=23127527), [Luo H](http://www.ncbi.nlm.nih.gov/pubmed?term=Luo%20H%5BAuthor%5D&cauthor=true&cauthor_uid=23127527). Diagnostic value of EUS elastography in differentiation of benign and malignant solid pancreatic masses: a meta-analysis. Pancreatology. 2012;12:402-8.

45. Fuccio L, Hassan C, Laterza L, [Correale L](http://www.ncbi.nlm.nih.gov/pubmed?term=Correale%20L%5BAuthor%5D&cauthor=true&cauthor_uid=23660563), [Pagano N](http://www.ncbi.nlm.nih.gov/pubmed?term=Pagano%20N%5BAuthor%5D&cauthor=true&cauthor_uid=23660563), [Bocus P](http://www.ncbi.nlm.nih.gov/pubmed?term=Bocus%20P%5BAuthor%5D&cauthor=true&cauthor_uid=23660563), et al. The role of K-ras gene mutation analysis in EUS-guided FNA cytology specimens for the differential diagnosis of pancreatic solid masses: a meta-analysis of prospective studies. Gastrointest Endosc. 2013;78:596-608.

46. Kwee RM, Kwee TC. The accuracy of endoscopic ultrasonography in differentiating mucosal from deeper gastric cancer. Am J Gastroenterol. 2008;103:1801-9.

47. Hudelist G, English J, Thomas AE, Tinelli A, Singer CF, Keckstein J. Diagnostic accuracy of transvaginal ultrasound for non-invasive diagnosis of bowel endometriosis: systematicreview and meta-analysis. Ultrasound Obstet Gynecol. 2011;37:257-63.

48. Meredith SM, Sanchez-Ramos L, Kaunitz AM. Diagnostic accuracy of transvaginal sonography for the diagnosis of adenomyosis: systematic review andmetaanalysis. Am J Obstet Gynecol. 2009;201:107.e1-6.

49. Scarpa M, Bertin M, Ruffolo C, , [Polese L](http://www.ncbi.nlm.nih.gov/pubmed?term=Polese%20L%5BAuthor%5D&cauthor=true&cauthor_uid=18668671), [D'Amico DF](http://www.ncbi.nlm.nih.gov/pubmed?term=D'Amico%20DF%5BAuthor%5D&cauthor=true&cauthor_uid=18668671), [Angriman I](http://www.ncbi.nlm.nih.gov/pubmed?term=Angriman%20I%5BAuthor%5D&cauthor=true&cauthor_uid=18668671). A systematic review on the clinical diagnosis of gastrointestinal stromal tumors. J Surg Oncol. 2008;98:384-92.

50. Puli SR, Kalva N, Bechtold ML, , [Pamulaparthy SR](http://www.ncbi.nlm.nih.gov/pubmed?term=Pamulaparthy%20SR%5BAuthor%5D&cauthor=true&cauthor_uid=23801872), [Cashman MD](http://www.ncbi.nlm.nih.gov/pubmed?term=Cashman%20MD%5BAuthor%5D&cauthor=true&cauthor_uid=23801872), [Estes NC](http://www.ncbi.nlm.nih.gov/pubmed?term=Estes%20NC%5BAuthor%5D&cauthor=true&cauthor_uid=23801872), et al. Diagnostic accuracy of endoscopic ultrasound in pancreatic neuroendocrine tumors: a systematic review andmeta analysis. World J Gastroenterol. 2013;19:3678-84.

51. Angela E. Li, Bob T. Li, Bernard H. K. Ng, Sam McCormack, John Vedelago, et al. Diagnostic accuracy of imaging modalities in the evaluation of vascular invasion in pancreatic adenocarcinoma: A meta-analysis. World Journal of Oncology. 2013;4:74-82.

52. Teng J, Chen M, Gao Y, Yao Y, Chen L, Xu D. Transrectal sonoelastography in the detection of prostate cancers: a meta-analysis. BJU Int. 2012;110:E614-20.

53. Madhoun MF, Wani SB, Rastogi A, Early D, Gaddam S, Tierney WM, et al. The diagnostic accuracy of 22-gauge and 25-gauge needles in endoscopic ultrasound-guided fine needleaspiration of solid pancreatic lesions: a meta-analysis. Endoscopy. 2013;45:86-92.

54. Kwok H, Bissett IP, Hill GL. Preoperative staging of rectal cancer. Int J Colorectal Dis. 2000;15:9-20.

55. Ledro-Cano D. Suspected choledocholithiasis: endoscopic ultrasound or magnetic resonance cholangio-pancreatography? Asystematic review. Eur J Gastroenterol Hepatol. 2007;19:1007-11.

56. Petrov MS, Savides TJ. Systematic review of endoscopic ultrasonography versus endoscopic retrograde cholangiopancreatography forsuspected choledocholithiasis. Br J Surg. 2009;96:967-74.

57. Garrow D, Miller S, Sinha D, [Conway J](http://www.ncbi.nlm.nih.gov/pubmed?term=Conway%20J%5BAuthor%5D&cauthor=true&cauthor_uid=17478348), [Hoffman BJ](http://www.ncbi.nlm.nih.gov/pubmed?term=Hoffman%20BJ%5BAuthor%5D&cauthor=true&cauthor_uid=17478348), [Hawes RH](http://www.ncbi.nlm.nih.gov/pubmed?term=Hawes%20RH%5BAuthor%5D&cauthor=true&cauthor_uid=17478348), et al. Endoscopic ultrasound: a meta-analysis of test performance in suspected biliary obstruction. Clin Gastroenterol Hepatol. 2007;5:616-23.

58. Xu W, Shi J, Li X, [Zeng X](http://www.ncbi.nlm.nih.gov/pubmed?term=Zeng%20X%5BAuthor%5D&cauthor=true&cauthor_uid=23169307), [Lin Y](http://www.ncbi.nlm.nih.gov/pubmed?term=Lin%20Y%5BAuthor%5D&cauthor=true&cauthor_uid=23169307). Endoscopic ultrasound elastography for differentiation of benign and malignant pancreatic masses: a systemic review and meta-analysis. Eur J Gastroenterol Hepatol. 2013;25:218-24.

59. De Lisi S, Leandro G, Buscarini E. Endoscopic ultrasonography versus endoscopic retrograde cholangiopancreatography in acute biliary pancreatitis: a systematic review. Eur J Gastroenterol Hepatol. 2011;23:367-74.

60. Wu LM, Jiang XX, Gu HY, [Xu X](http://www.ncbi.nlm.nih.gov/pubmed?term=Xu%20X%5BAuthor%5D&cauthor=true&cauthor_uid=21183858), [Zhang W](http://www.ncbi.nlm.nih.gov/pubmed?term=Zhang%20W%5BAuthor%5D&cauthor=true&cauthor_uid=21183858), [Lin LH](http://www.ncbi.nlm.nih.gov/pubmed?term=Lin%20LH%5BAuthor%5D&cauthor=true&cauthor_uid=21183858), et al. Endoscopic ultrasound-guided fine-needle aspiration biopsy in the evaluation of bile duct strictures andgall bladder masses: a systematic review and meta-analysis. Eur J Gastroenterol Hepatol. 2011;23:113-20.

61. Smith-Bindman R, Kerlikowske K, Feldstein VA, [Subak L](http://www.ncbi.nlm.nih.gov/pubmed?term=Subak%20L%5BAuthor%5D&cauthor=true&cauthor_uid=9809732), [Scheidler J](http://www.ncbi.nlm.nih.gov/pubmed?term=Scheidler%20J%5BAuthor%5D&cauthor=true&cauthor_uid=9809732), [Segal M](http://www.ncbi.nlm.nih.gov/pubmed?term=Segal%20M%5BAuthor%5D&cauthor=true&cauthor_uid=9809732),  et al. Endovaginal ultrasound to exclude endometrial cancer and other endometrial abnormalities. JAMA.1998;280:1510-7.

62. Toloza EM, Harpole L, McCrory DC. Noninvasive staging of non-small cell lung cancer: a review of the current evidence. Chest. 2003;123:137S-46S.

63. Hariharan D, Constantinides V, Kocher HM, Tekkis PP. The role of laparoscopy and laparoscopic ultrasound in the preoperative staging of patients with resectable colorectal liver metastases: a meta-analysis. Am J Surg. 2012;204:84-92.

64. Puli SR, Bechtold ML, Buxbaum JL, Eloubeidi MA. How good is endoscopic ultrasound-guided fine-needle aspiration in diagnosing the correct etiology for a solid pancreatic mass?: A meta-analysis and systematic review. Pancreas. 2013;42:20-6.

65. Toloza EM, Harpole L, Detterbeck F, McCrory DC. Invasive staging of non-small cell lung cancer: a review of the current evidence. Chest. 2003;123:157S-66S.

66. Zhou Y, Chen X. Comparison with EUS and ERCP diagnosis of common bile duct stones:A Meta-analysis. Chinese journal of gastroenterology and hepatology. 2008;12:129-32

67. Zhu CL, Huang Q, Liu CH, Xie F, Yao Q. Diagnostic value of endoscopic ultrasound-guided ﬁne-needle aspiration for solid pancreatic masses: A meta-analysis. World Journal of Gastroenterology. 2012;20:1667-76

68. Hu DM, Gong TT, Zhu Q. Contrast-enhanced EUS indifferential diagnosis of benigh and malignant pancreatic masses: a meta-analysis. Chinese Journal of Digestive Endoscopy. 2012;29:374-9

69. Si YF. Accuracy of endoscopic ultrasound on prominence lesions of gastrointestinal tract: A Systematic Review. China Healthcare and Nutritio. 2012;10:4152-3.

70. LiN, WenYH, GuoHM. Accuracy of endoscopic ultrasound in the preoperative TN staging for gastric cancer: A Meta-analysis.Modern Oncology. 2012;20:110-4

71. ZhuLL, WangYP. MRCP versus EUS for diagnosis of bile ductobstruction:A Systematic Review. Chinese Journal of Digestive Endoscopy. 2012;30:154-9

72. Huang XF, Han CN, Lin KQ,  [Zhang J](http://www.ncbi.nlm.nih.gov/pubmed?term=Zhang%20J%5BAuthor%5D&cauthor=true&cauthor_uid=20646538), [Xu H](http://www.ncbi.nlm.nih.gov/pubmed?term=Xu%20H%5BAuthor%5D&cauthor=true&cauthor_uid=20646538), [Zhang XM](http://www.ncbi.nlm.nih.gov/pubmed?term=Zhang%20XM%5BAuthor%5D&cauthor=true&cauthor_uid=20646538). Meta-analysis of ultrasonography in diagnosis of deeply infiltrating endometriosis. Chinese Journal of Obstetrics and Gynecology. 2010;45:269-72.
